# Metabolites of L-ARG in Exhaled Breath Condensate and Serum Are Not Biomarkers of Bronchial Asthma in Children

**DOI:** 10.3390/jcm11010252

**Published:** 2022-01-04

**Authors:** Joanna Połomska, Barbara Sozańska

**Affiliations:** 1st Department and Clinic of Paediatrics, Allergology and Cardiology, Wrocław Medical University, 50-367 Wrocław, Poland; barbara.sozanska@umw.edu.pl

**Keywords:** asthma, L-arginine, ADMA, EBC

## Abstract

(1) Background: L-arginine (L-ARG) and its metabolites are involved in some aspects of asthma pathogenesis (airway inflammation, oxidative stress, bronchial responsiveness, collagen deposition). Published data indicate that lungs are a critical organ for the regulation of L-ARG metabolism and that alterations in L-ARG metabolism may be significant for asthma. The aim of this study was to assess the levels of L-ARG and its metabolites in pediatric patients with asthma in serum and exhaled breath condensate (EBC) by mass spectrometric analysis and compare them with non-asthmatic children. (2) Methods: Sixty-five children (37 pediatric patients with bronchial asthma and 28 healthy control subjects) aged 6–17 participated in the study. All participants underwent a clinical visit, lung tests, allergy tests with common aeroallergens, and serum and EBC collection. The levels of biomarkers were determined in both serum and EBC. Analytical chromatography was conducted using an Acquity UPLC system equipped with a cooled autosampler and an Acquity HSS T3 column. Mass spectrometric analysis was conducted using the Xevo G2 QTOF MS with electrospray ionization (ESI) in positive ion mode. (3) Results: Asymmetric dimethylarginine (ADMA) and symmetric dimethylarginine (SDMA) levels in serum and EBC did not differ significantly in asthmatic children and healthy control subjects. We found no correlation between forced expiratory volume in one second (FEV1) and L-ARG and its metabolites, as well as between interleukin-4 (IL-4) serum level and L-ARG and its metabolites. Concentrations of ADMA, SDMA, citrulline (CIT), and ornithine (ORN) were higher in serum than EBC in asthmatics and non-asthmatics. By contrast, concentrations of dimethylarginine (DMA) were higher in EBC than serum. ADMA/L-ARG, SDMA/L-ARG, and DMA/L-ARG ratios were significantly higher in EBC than in serum in asthmatics and in non-asthmatics. (4) Conclusions: Serum and EBC concentrations of L-ARG and its metabolites were not an indicator of pediatric bronchial asthma in our study.

## 1. Introduction

We presently know that asthma in children is a collection of different phenotypes that are associated with variable disease courses, and their inflammatory patterns are not completely characterized [1]. The biological samples that provide information on asthma-related inflammation are not easy to obtain from the respiratory tract because procedures such as bronchoalveolar lavage or bronchoscopy with bronchial biopsy are too invasive for routine use [2]. Several studies in human asthma and in animal models of allergic airway inflammation suggest that alterations in L-ARG and nitric oxide (NO) metabolic pathways in the lung may be involved in the pathophysiology of bronchial asthma [3,4,5,6,7]. L-ARG, a conditionally essential amino acid, is required in the biosynthesis of proteins and serves as an important substrate for two distinct groups of enzymes: the nitric oxide synthase family (NOS) and arginases [8]. The lungs have an important role in the regulation of L-ARG metabolism through the production and degradation of methylarginines [9]. The reaction of methylation protein-incorporated arginine residues is catalyzed by a family of enzymes called protein arginine methyltransferases (PRMT) [9]. Free forms of methylarginine have been identified: monomethylarginine (MMA), ADMA, and SDMA [9].

The biosynthesis of NO from L-ARG by the NOS plays an important role in asthma, including bronchodilatation by relaxing airway smooth muscle [10]. L-ARG metabolism is significantly altered in asthma due to increased expression of inducible NOS (iNOS) [11,12]. However, overproduction of NO has been indicated to contribute to tissue injury by its reaction with superoxide and then peroxynitrite production and increased oxidative stress [8]. Prolonged oxidative stress seems to promote also the accumulation of ADMA in the lungs [13,14]. ADMA serves as an endogenous competitive inhibitor of both constitutive forms (neuronal NOS, endothelial NOS) and inducible forms of NOS [15]. SDMA impairs the cellular uptake of L-ARG and thus may potentially reduce intracellular substrate bioavailability [15]. More recently, focus has turned to the pathway of L-ARG metabolism by arginase, which reduces the bioavailability of endogenous L-ARG for NOS [16]. In animal models, arginase activity in the lungs may be induced by Th2 cytokines, such as IL-4 and interleukine-13 (IL-13), produced at elevated levels in asthmatic airways and activating inflammatory pathways [17]. Arginase may be involved in asthma pathogenesis via mechanisms such as the modulation of NOS activity, the reduction of L-ARG bioavailability, and increased ORN generation [16].

Monitoring of airway inflammation on the basis of biomarkers in serum and EBC would give a direct insight into the underlying pathological processes in pediatric bronchial asthma. EBC is a non-invasive method for the collection of airway secretions and is also suitable for use in children [18,19]. However it remains unknown as to whether biomarker levels in EBC correlate with those measured in serum. Published data on the role of L-ARG and its metabolites in pediatric bronchial asthma are limited and clinical applications for the assessment of the concentration of these biomarkers in different biological samples (e.g., serum, EBC) are not currently clear. The role of L-ARG and its metabolites as indirect biomarkers to identify asthmatic patients in clinical practice is unknown.

Therefore, the aims of the study were to evaluate the levels of L-ARG, ADMA, SDMA, CIT, ORN, and DMA in pediatric patients with asthma in the serum and EBC by mass spectrometric analysis and to determine the relationship between these markers in both locations to better understand their role in pediatric asthma pathology.

## 2. Materials and Methods

### 2.1. Participants

Sixty-five children aged 6–17 were invited to take part in the study. The study group included 37 children (25 boys, 12 girls) with a diagnosis of atopic or non-atopic bronchial asthma. The children were randomly recruited from patients at the Department of Pediatric Allergology or the Pediatric Allergology Outpatient Clinic (Wroclaw Medical University, Poland). Questionnaires, designed for the purposes of this study, included information about children’s socio-demographic characteristics, health status, and ongoing treatment and were completed for all the participants. The diagnosis of asthma was established by a physician and based on typical symptoms (wheezing, shortness of breath, cough, and chest tightness), clinical history, results of functional lung tests, and positive response to a bronchodilator. The control group included 28 people (16 boys, 12 girls) with no history of bronchial asthma or any other allergic disease.

Exclusion criteria for the study were: refusal to participate in the study, mental retardation, birth defects of the respiratory system, heart defects, chronic kidney disease, active smoking, current allergen immunotherapy or immunotherapy in the previous 3 months, current biological treatment of asthma or biological treatment in the previous 3 months, current respiratory tract infection and respiratory tract infection in the 3 weeks preceding the study. An acute upper airway infection was understood as the presence of general symptoms: an increase in body temperature above 37.5 °C, malaise, and the presence of symptoms from the upper respiratory tract—acute onset of rhinitis/sore throat.

This study was approved by the Ethics Committee of the Wroclaw Medical University KB—554/2017. All the participants and their guardians received information regarding the background and the implementation of the study. Written informed consent was obtained from children and their parents. They were given the opportunity to withdraw from participation in the study at any time.

### 2.2. Medical History and Physical Examination

Medical history, physical condition, and medication taken were recorded. General medical examination was performed to exclude patients with a current respiratory tract infection or patients with skin changes that were contraindications to the skin prick test (SPT). Medical examination included anthropometric measurements (body weight, height).

### 2.3. Exhaled Breath Condensate Collection

In all participants, EBC was collected according to the European Respiratory Society (ERS)/American Thoracic Society (ATS) recommendations [18]. The RTube disposable collection system (Respiratory Research, Inc., USA) was used in the procedure and the tube was cooled by a cooling sleeve placed around it. The cooling sleeve was stored in a freezer (−30 °C) until needed. After applying a nose-clip, the child breathed normally for 10 min through a special mouthpiece. The time of sampling and the patient’s behavior during the procedure were supervised by the researcher. Immediately after collection was completed, the cartridge with the sample was placed in a heat-insulating bag, impermeable to light, and then delivered to the laboratory, transferred into Eppendorf tubes, and stored at −80 °C.

### 2.4. Baseline Functional Respiratory Test

Spirometry tests were performed (Jaeger/CareFusion, Germany). The subjects had to complete at least three forced vital capacity (FVC) maneuvers to satisfy the ERS/ATS guidelines [20]. The curve with the highest FVC and FEV1 values was selected for analysis.

### 2.5. Atopy Status

Allergic sensitization was evaluated by SPT or serum specific immunoglobulin E (IgE) measurements for the most common respiratory allergens. SPTs were performed by a researcher with commercial extracts of inhalant allergens: grass mix, common rye, warty birch pollen, common hazel, alder, mugwort, Dermatophagoides pteronyssinus, Dermatophagoides farinae, Alternaria alternata, cat dander, dog fur (HAL Allergy, The Netherlands), and with negative (saline) and positive (histamine) control solutions. Tests were applied to the volar aspect of the forearm, at least 2–3 cm from the wrist and the antecubital fossae. The location of each allergen was marked with a pen on the forearm to properly identify test results [21]. The largest diameter of the wheal of each test was measured and skin prick tests were considered to be positive if they induced a diameter of ≥ 3 mm [22]. In patients with a history of measurement of specific IgE antibodies in the last year, the available data were used instead of the skin prick test.

### 2.6. Blood Sample Collection, Storage, and Preparation

Blood samples were obtained in the morning of the examination after an overnight fast. Venous blood was collected from each participant from the cubital vein into S-Monovette Sarstedt tubes. After centrifugation, the serum was separated from the blood clot. Serum in a volume of approximately 2 mL from each patient was divided into 2 portions of approximately 1 mL into Eppendorf tubes and stored until the day of determination under the following conditions: the serum samples for the determination of IL-4 were stored at −20 °C, and the serum samples for the determination of L-ARG and its metabolites were stored at −80 °C.

### 2.7. Interleukin-4 Concentrations

The serum levels of IL−4 were determined by Quantikine HS Human IL-4 (ELISA; Quantikine HS Human IL-4, R&D Systems, Minneapolis, USA) using the method recommended by the manufacturer. The intensity of staining was read with a Biotech ELX800 plate reader and expressed in pg/mL. Individual samples were measured twice and the mean values were further analyzed.

### 2.8. L-ARG, ADMA, SDMA, CIT, ORN, and DMA Concentrations

Samples were prepared based on the method that was previously published [23].

The following steps were performed:

1. 100 µL aliquots of calibration standards or 100 µL of serum, 10 µL of internal standard solution (50 µM D6-DMA, 20 µM D7-ADMA, 100 µM D6-ornithine, and 100 µM D7-arginine, respectively), and 50 µL of borate buffer (0.025 M Na2B4O7 × 10H2O, 1.77 mM NaOH, pH = 9.2) were added into 2.0 mL polypropylene tubes and vortexed (1 min, 25 °C).
2. Derivatization was performed using 400 µL of acetonitrile (ACN) and 10 µL of 10% BCl in ACN. The samples were incubated and vortexed (5 min, 25 °C), and centrifuged (7 min, 17,000 RCF, 4 °C). After this, 100 µL of the clear supernatant was transferred into glass tubes containing 400 µL of water.
3. Preparation of standard calibration curves was performed using the following concentration ranges: 5; 12.5; 25; 50; 100; 150; 200; 250 µM for L-ARG, 0.05; 0.13; 0.25; 0.5; 1.0; 1.5; 2; 2.5 µM for ADMA and SDMA, 1; 2.5; 5; 10; 20; 30; to 50 µM for CIT, 2.5; 7.5; 15; 30; 60; 90; 120; 150 µM for ORN, and 0.14; 0.35; 0.7; 1.4; 2.8; 4.2; 5.6; 7.0 µM for DMA.
4. Analytical chromatography was performed using an Acquity UPLC system containing a cooled autosampler (Waters, Milford, MA, USA) and Acquity HSS T3 column (50 × 1.0 mm, 1.75 µm) from Waters. Elution was conducted with 0.1% formic acid (FA) in water as mobile phase A and 0.1% FA in methanol as mobile phase B. The volume was 2 µL. Total run time was 7 min, with a total flow rate of 250 µL/min. The following gradient was used: 5% B for 0–0.5 min, 5–14% B for 0.5–3 min, 14–60% B for 3–4 min, 60–90% B for 4–4.5 min, 90% B for 4.5–5 min and 90–5% B for 5–5.10 min.
5. Mass spectrometric analysis was conducted using Xevo G2 QTOF MS (Waters, Milford, MA, USA) with ESI in positive ion mode. Parameters such as the spray voltage, source temperature, and the desolvation temperature were set at 0.5 kV, 120 °C, and 450 °C, respectively. Nitrogen was used as the nebulizing and drying gas. Data were acquired by using MassLynx software (Waters, Milford, MA, USA) for the following ions (m/z): 279.1457 (for L-ARG), 286.1749 (for D7-arginine), 307.1717 (for ADMA and SDMA), 314.2076 (for D7-ADMA), 280.1297 (for CIT), 341.1501 (for ORN), 347.1878 (for D6-ornithine), 150.0919 (for DMA), and 156.1113 (for D6-DMA).

The samples of EBC were analyzed according to the same method as serum samples, with a modification involving additional sample drying steps before and after the derivative reaction of the analytes.

### 2.9. Statistical Analysis

The analyses were performed with STATISTICA v. 13.3 (TIBCO Software Inc) and EXCEL. The mean values (M), standard deviations (SD), median (Me), lower quartiles (Q1), and upper (Q3), as well as the variability range (Min) and (Max), were calculated for all quantitative traits. The assessment of the compatibility of empirical distributions with the theoretical normal distribution was checked by the Shapiro–Wilk test. Qualitative features are shown in the tables in the form of numbers (n) and fractions (%). Correlations between quantitative variables were expressed as Spearman’s correlation coefficient. In situations in which the quantitative data were not normally distributed, a nonparametric Wilcoxon rank sum test was used. In all tests, values of *p*  <  0.05 were deemed to be statistically significant.

## 3. Results

### 3.1. Characteristics of Participants

We studied 37 asthmatics (12 girls, 25 boys, mean age 10.9 ± 2.7 years) and 28 healthy controls (12 girls, 16 boys, mean age 12.6 ± 2.5 years). The two groups were similar in terms of living conditions (contact with dog/cat in home, exposure to tobacco smoke) and lifestyle (participation in physical education classes), but the asthmatic group was younger than the control group. There were also differences regarding body mass index (BMI). As expected, pulmonary function based on FEV1 was lower in the asthmatic group. The characteristics of all the participants are presented in Table 1.

Median asthma duration was over four years and most of the asthmatic children were also atopic. The detailed asthma and allergic status characteristics of the study group are presented in Table 2.

### 3.2. Biochemical Analyses

Biological samples were obtained from all 65 participants (37 asthmatics and 28 healthy control subjects).

Serum concentrations of L-ARG and its metabolites: Due to a pre-laboratory error, three (-3) of the blood samples were lost. For further analysis, measurable serum concentrations of L-ARG and its metabolites were found in 62 samples that were evaluated in the study (36 asthmatics, 26 non-asthmatics). The mean serum L-ARG, ADMA, SDMA, CIT, ORN, and DMA were similar in the asthmatic and control groups (Table 3).

Serum concentrations of IL-4: One (−1) of the serum samples was lost due to a pre-laboratory error. For further analysis, measurable serum concentrations of IL-4 were found in 64 samples that were evaluated in the study (37 asthmatics, 27 non-asthmatics). Regarding the mean serum IL-4 concentration, there were no significant differences among the two groups. Analysis of the correlation between serum and EBC L-ARG metabolites and the serum level of IL-4 did not reveal any relationship (Table 4).

EBC concentrations of L-ARG and its metabolites: Ten (−10) of the EBC samples were used for the pilot study to test the sensitivity of the EBC analysis method (5 samples from study group and 5 from control group); two (−2) EBC samples were lost due to a pre-laboratory error and another (−1) sample of EBC was used to calibrate the method. Using the RTube device among the pediatric population resulted in 400–750 µg of EBC material after 10 min of recommended sampling time collection. Measurable EBC concentrations of L-ARG and its metabolites were found in 52 samples that were evaluated in the study (52 samples, 32 asthmatics, 20 non-asthmatics). There were no differences in EBC concentrations of L-ARG ADMA, SDMA, CIT, ORN, and DMA in the EBC of asthmatics and healthy controls (Table 5). Regarding the L-ARG metabolites/L-ARG ratio, in the EBC, the DMA/L-ARG ratio was almost two hundred times higher than in serum.

**Figure 1:**
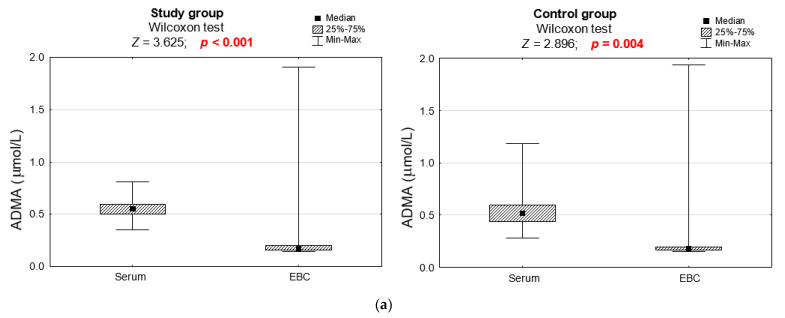

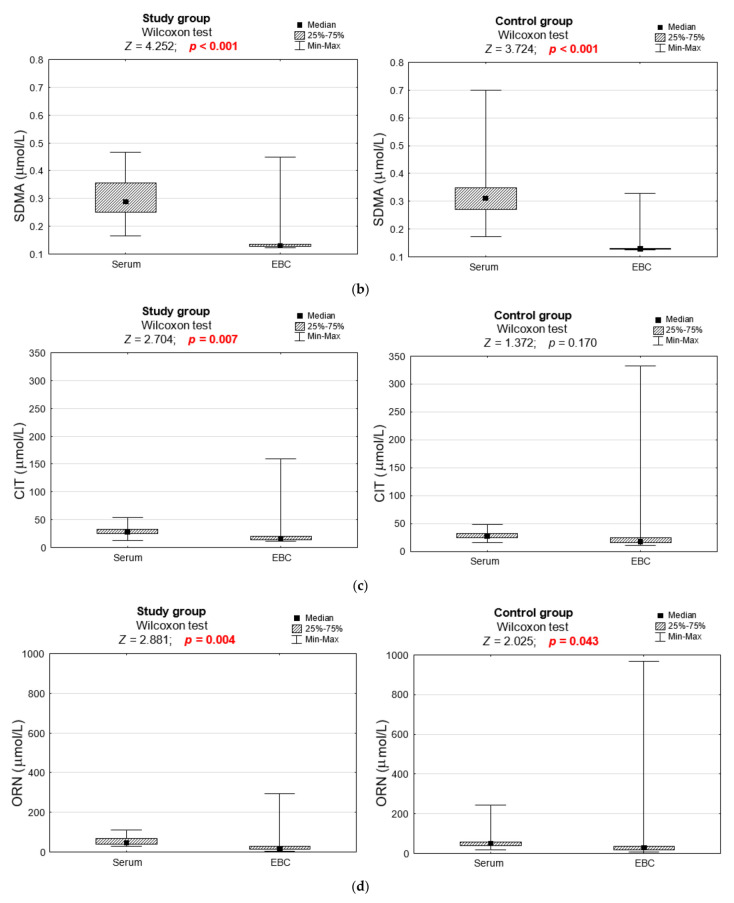

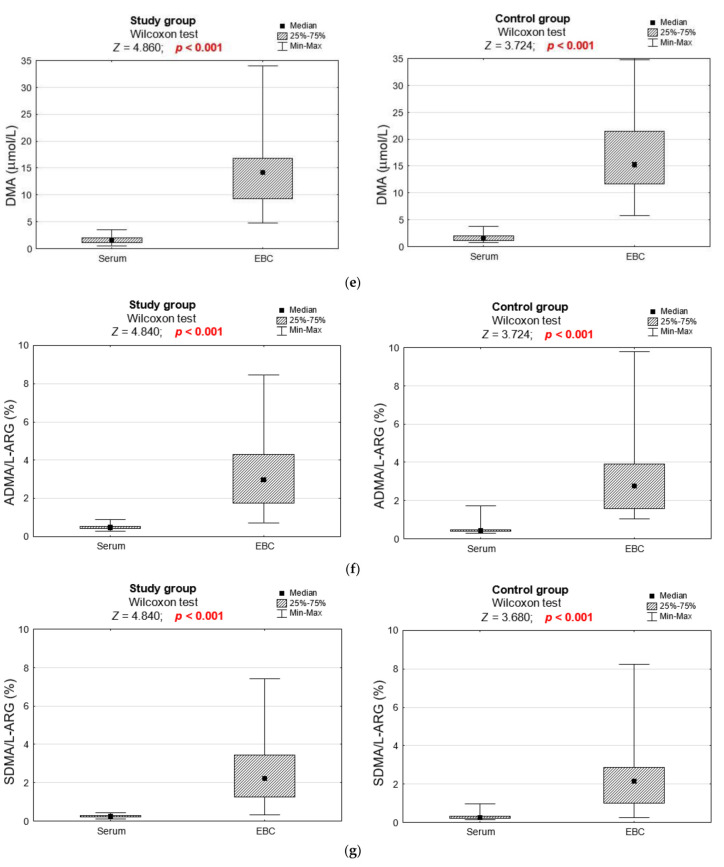

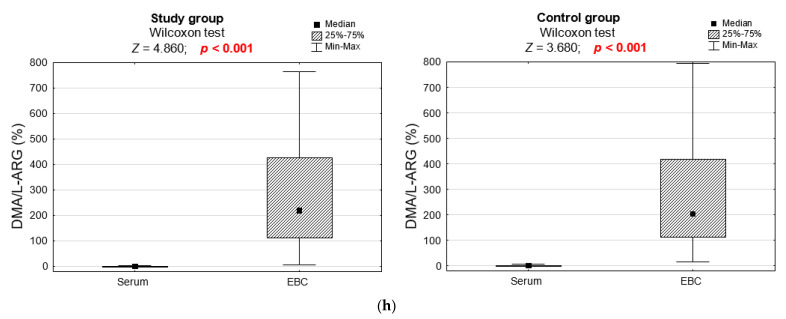
(**a**–**h**) Comparison of the concentrations of L-ARG and its metabolites in serum and EBC between study group and control group. The significance of differences in the assessment of L-ARG and its metabolites in serum and EBC of asthmatic and non-asthmatic subjects was verified using the Wilcoxon test.

Concentrations of ADMA, SDMA, CIT, and ORN were higher in serum than EBC in asthmatics and non-asthmatics. By contrast, concentrations of DMA were higher in EBC than serum. ADMA/L-ARG, SDMA/L-ARG, and DMA/L-ARG ratios were significantly higher in EBC than in serum in asthmatics and in non-asthmatics (Figure 1).

Correlation comparisons were made between L-ARG and its metabolites in serum and EBC measurements and parameters such as FEV1 and serum IL-4 concentration. There was no correlation between the analyzed parameters. All correlation coefficients did not differ from zero (*p* > 0.05) (Table 6).

Additional statistical analysis was performed to evaluate the correlation between biochemical measurements and clinical parameters such as age, BMI, atopy status, and treatment in asthmatics (see Supplementary Materials: Tables S1–S8). Regarding the mean serum (Table S1) and EBC (Table S2) concentrations of L-ARG, ADMA, SDMA, CIT, ORN, and DMA, there were no significant differences among asthmatics aged 6–11 years and asthmatics aged 12–17 years. There was no correlation found in the BMI of asthmatics and serum (Table S3) or EBC (Table S4) concentrations of L-ARG and its metabolites. All correlation coefficients did not differ from zero (*p* > 0.05). Data were also analyzed to compare atopic and non-atopic asthmatics. Regarding the mean serum (Table S5) or EBC (Table S6) concentrations of L-ARG, ADMA, SDMA, CIT, ORN, and DMA, there were no significant differences among atopic and non-atopic asthmatics. Analysis conducted to compare patients treated with inhaled corticosteroids in the previous 4 weeks and patients who had no ICS treatment in the previous 4 weeks did not reveal statistically significant differences between the two analyzed groups (Tables S7 and S8).

## 4. Discussion

In the present study, we investigated the metabolic pathways of L-ARG and its metabolites in asthmatic children and healthy controls, and we demonstrated that ADMA and SDMA levels both in serum and EBC did not differ in asthmatic children and healthy control subjects. In our study, we also found no correlation between FEV1 and L-ARG metabolites as well as between IL-4 serum level and L-ARG metabolites. Concentrations of metabolites were higher in serum than EBC, both in asthmatics and non-asthmatics. Concentrations of DMA, however, were higher in EBC than serum. ADMA/L-ARG, SDMA/L-ARG, and DMA/L-ARG ratios were significantly higher in EBC than in serum in both studied groups.

It has been postulated in previous studies that ADMA might play an important role in the regulation of the L-ARG/NO pathway in asthma. In support of this, L-ARG and its metabolites have been evaluated in experimental models of asthma, and it was reported that, in murine models, ADMA may be involved in the pathogenesis of asthmatic airway inflammation [13]. Ahmad et al. demonstrated that, in murine models, ADMA metabolism is upregulated in inflamed bronchial epithelial cells and ADMA levels correlate with reactive oxygen species production [24]. Wells et al. observed that the administration of ADMA reduced nitrite production while increasing superoxide levels in a dose-dependent manner [13]. Wells et al. also suggested that ADMA may affect lung function and airway hyperreactivity even in non-inflamed airways and be associated with collagen production and deposition [13]. Taken together, these animal studies support the notion that ADMA might act as an important factor related to lung inflammation in asthma.

Previous studies on serum and plasma concentrations of L-ARG metabolites in asthmatic patients gave inconsistent results. In a study by Lara et al., plasma concentrations of L-ARG, ORN, CIT, ADMA, and SDMA were not significantly different among the asthmatic group and healthy group (*n* = 232 with both non-severe and severe asthma, including 30 asthmatic children aged 6–17 years, *n* = 26 for healthy control group). However, subjects with asthma were found to have greater L-ARG bioavailability (evaluated by the ratio L-ARG to products of enzymatic catabolism L-ARG/[ORN + CIT]) compared with healthy control subjects [25]. In another study, high plasmatic levels of ADMA in asthmatic subjects were reported. They were inversely correlated with BMI, suggesting a role of this biomarker in the physiopathology of the late-onset asthma phenotype [6]. In contrast, Riccioni et al. observed that mean plasma ADMA, SDMA, and L-ARG concentrations were significantly lower in children with at least a 2-year history of mild persistent allergic bronchial asthma with respect to the control group (*n* = 50 for asthmatic group and *n* = 10 for healthy controls) [4]. Kraj et al. also observed decreased serum L-ARG levels in asthmatic children (*n* = 30 for asthmatic group and *n* = 20 for healthy controls) [26]. The largest study to examine the ADMA concentration in serum and its relationship with asthma was conducted by Lau et al., and the study population included 314 children (age = 8 years, *n* = 72 for current asthma, *n* = 242 no current asthma). In this study, children with asthma had a mild phenotype, as evidenced by their well-preserved lung function. The results of the study appeared to contrast the available human data, because the authors reported that L-ARG levels were not significantly different between children with and without asthma and the L-ARG/ADMA ratio was also not significantly different between asthmatic children and the control group. This study did not support the view that systemic levels of ADMA or L-ARG are associated with asthma or airway inflammation in children [27].

As shown above, recent studies on L-ARG and its metabolites in asthma showed different results. However, it is difficult to compare the outcomes of these investigations due to the large heterogeneity in the study population and study design. There were various asthma phenotypes studied, including non-atopic and atopic asthma, mild to severe state, and different age groups. Moreover, respiratory specimens were not collected in these studies. Serum measures might not reflect pulmonary or intracellular levels and might not reflect the airway pathological process in bronchial asthma. One of the key issues is understanding the distribution of L-ARG and its metabolites between serum and the lung compartment in asthmatic patients.

Scott et al. revealed higher levels of ADMA and SDMA in human adult autoptic lung tissue (*n* = 6 for control group and *n* = 5 for asthma group) and sputum samples collected from pediatric patients with bronchial asthma (*n* = 17 for asthma group, median age 12 years, all treated with inhaled corticosteroids, *n* = 12 for control group, median age 18.5 years). The authors demonstrated that ADMA and SDMA levels were increased in the lungs of subjects with asthma, compared with the control group. However, SDMA concentrations were decreased in sputum samples from pediatric patients with asthma [3].

Data on L-ARG metabolite concentrations are scarce. Carraro et al. measured ADMA in EBC (*n* = 77 asthmatic children, *n* = 65 healthy children) and serum obtained from asthmatic children (*n* = 28 asthmatic children). In their study, ADMA levels in the EBC of asthmatic children were significantly higher than in the healthy control subjects but EBC ADMA levels did not correlate with those measured in serum. The authors concluded that the serum ADMA level might not be reflective of pathological processes in the lung. The authors did not find any correlation between levels of ADMA in EBC and lung function parameters [5].

The findings of our study complement earlier studies. To the best of our knowledge, our study appears to be the first report to assess L-ARG metabolism in serum and EBC (i.e., L-ARG, ADMA, and SDMA), functional outcomes (FEV1), and serum IL-4 concentrations in asthmatic children and compare them to children with no current asthma. Whilst our study did not confirm all the results obtained by Carraro et al., it did substantiate the notion that L-ARG and its metabolites may be evaluated in airway specimens such as EBC. In Carraro’s study, EBC was collected using TURBO-DECCS and the absolute median ADMA concentration in EBC was 1.6 pmol/mL. Our findings cannot be directly compared with Carraro’s investigations of EBC ADMA concentrations, and the first potential reason could be the fact that, for the collection of EBC, different condenser systems were used. Carraro et al. used a commercially available collection system that included an electrical cooling system and allowed them to adjust the temperature of sampling [28]. In our study, we chose to use disposable single-patient equipment to collect airway specimens (RTube). The construction of this condensing equipment did not allow us to maintain the sampling at a constant low temperature. The pre-cooled sleeve of RTube is sensitive to higher ambient temperatures and, during the procedure, the temperature gradually increases, which therefore limits the time of effective collection [29]. The temperature of sampling may potentially affect the composition of EBC [19]. Our mean ADMA concentration in EBC was 0.31 µmol/L and we did not use the marker of dilution.

The next reason is that, in Carraro’s study, there were no ADMA serum measurements in the healthy control subjects. In our investigation, ADMA was measured in the serum of the children with asthma and in healthy children and the mean concentrations were 0.56 µM/L and 0.54 µM/L, respectively, and these measurements were similar to results obtained by Carraro in the serum of children with asthma—mean 0.53 µM—supporting the reproducibility of the serum measurements.

The discrepancy between our results and other studies on L-ARG and ADMA in the serum of children with asthma may be explained by the influence of the acute exacerbation of the disease, and it is conceivable that systemic L-ARG metabolism may alter during exacerbations. The asthmatic population enrolled by Carraro had significantly lower (*p* < 0.001) spirometric parameters than healthy control subjects. In our study, median pulmonary function parameters differed in FEV1 (*p* = 0.04) and median FEV1 in asthmatic children was 97 (% predicted). A possible explanation for the different results could be also the influence of the use of treatment in asthmatics. Further studies with a prospective design are needed to explain whether inhaled steroid therapy, montelukast, or long-acting B-agonists affect the concentration of L-ARG metabolites in EBC. The role of ADMA in asthma pathogenesis may also differ between young children, adolescents, and adults. Thus, further studies in better-defined and homogenous groups of asthmatic subjects are desirable to clarify whether alterations in the L-ARG metabolome may be informative for asthma.

In this study, we showed that serum levels of ADMA, SDMA, CIT, and ORN are higher than those measured in samples of EBC. Interestingly, DMA concentrations were significantly higher in EBC than in serum. ADMA and SDMA are produced by the post-translational methylation of arginine residues in proteins and they are liberated during protein degradation [9]. A large fraction of ADMA, but not SDMA, is hydrolyzed by dimethylarginine dimethylaminohydrolaze (DDAH) to form DMA, which is excreted in urine. In our study, the ADMA/L-ARG, SDMA/L-ARG, and DMA/L-ARG ratios were significantly higher in EBC than in serum. It has been suggested that the lungs have an important role in the regulation of L-ARG metabolism by the production and clearance of methylarginines [9]. The underlying mechanisms warrant further investigations.

The strength of our study was the focus on a combination of non-invasive and minimally invasive methods (exhaled breath condensate, functional lung tests, blood tests) and a broad spectrum of tested markers. Another strength of this study was the uniform study protocol—we obtained all the measurements in asthmatic children and in healthy control pediatric subjects. To our knowledge, this is the first study comparing ADMA and other L-ARG metabolite measurements using a portable RTube collector to obtain lung specimens, and we demonstrated that measurable levels of biomarkers were observed in all samples obtained with this type of equipment. The collection of samples was successful in all children and the time of EBC sampling (10 min) was accepted by all children without loss of interest.

We recognize that a limitation of our study is that it was based on a small sample of participants. An additional limitation is the heterogeneity of our population, especially regarding age and BMI. It would have been perfect if the asthmatic patients and non-asthmatic subjects had been uniform in terms of age and BMI. However, we performed additional analysis to assess the correlation between concentrations of L-ARG and its metabolites (in serum and EBC of asthmatics) and clinical parameters such as age, BMI, atopy status, and treatment and, in our study, differences were not statistically significant. This study was also limited by the absence of the marker of dilution of the EBC. It should also be noted that, in our study, salivary contamination was not detected in the EBC samples. This weakness is partly balanced by the fact that, according to our knowledge, our study was the first one in which such a broad spectrum of biomarkers was evaluated in the serum and EBC of asthmatic children and compared to non-asthmatic subjects, and we used a collection device that would potentially attract the attention of researchers because of its simple construction and single-use design

## 5. Conclusions

In conclusion, our findings indicate that exhaled breath condensate sampling in a non-invasive way is a potential method for studying L-ARG and its metabolites. In our study, there were no significant differences between both serum and EBC concentrations of L-ARG and its metabolites in children with bronchial asthma with respect to the control group. However, in our opinion, the differences among different study results may be connected with different asthma phenotypes or endotypes. If L-ARG/ADMA-mediated changes in NO metabolism would be, in fact, a metabolic pathway of a specific asthma phenotype, it may have significant clinical implications and lead to new phenotype-specific therapeutic options. We hope that our study will help in the design of further studies to investigate asthma biomarkers in airway specimens such as exhaled breath condensate to explore the role of ADMA in asthma pathogenesis.

## Figures and Tables

**Table 1:** Patients’ characteristics.

Characteristic	Study Group *n* = 37	Control Group *n* = 28	*p*
Gender, *n* (%):			0.547
Girls	12 (32.4%)	12 (42.9%)	
Boys	25 (67.6%)	16 (57.1%)	
Age (years):			0.010
*M* ± SD	10.9 ± 2.7	12.6 ± 2.5	
*Me* [*Q*1; *Q*3]	10 [9; 13]	13 [12; 14]	
*Min*–*Max*	6–17	7–17	
Age groups, *n* (%):			0.002
6–11 years	25 (67.6%)	7 (25.0%)	
12–17 years	12 (32.4%)	21 (75.0%)	
Residential area:			0.291
Rural	10 (27.0%)	12 (42.9%)	
<15.000 residents	4 (10.8%)	1 (3.6%)	
>15.000 residents	23 (62.2%)	15 (53.6%)	
BMI (kg/m^2^):			0.012
*M* ± SD	18.6 ± 3.9	21.0 ± 3.4	
*Me* [*Q*1; *Q*3]	17 [16; 21]	21 [19; 23]	
*Min*–*Max*	12–29	15–30	
Number of siblings, *n* (%)			0.429
*M* ± SD	1.4 ± 1.1	1.3 ± 1.6	
*Me* [*Q*1; *Q*3]	1 [1; 2]	1 [1; 1]	
*Min*–*Max*	0–5	0–9	
Contact with an animal in the home environment:			
Dog	12 (32.4%)	10 (35.7%)	0.990
Cat	11 (29.7%)	10 (35.7%)	0.808
Exposure to tobacco smoke in the home	13 (35.1%)	12 (42.9%)	0.707
Participation in physical education classes	36 (97.3%)	28 (100.0%)	1.000
Pneumococcal vaccines	16 (43.2%)	7 (25.0%)	0.207
FEV1 (% predicted)	96.5 ± 15.0	104.8 ± 16.9	0.041
FVC (% predicted)	107.8 ± 15.0	110.6 ± 14.3	0.441
FEV1/FVC	89.3 ± 11.0	94.1 ± 10.2	0.075

*M*—Mean; *SD*—Standard deviation; *Me*—Median (50%); *Q*1—Lower quartile (25%); *Q*3—Upper quartile (75%); *Min*—Minimum; *Max*—Maximum; *n*—numbers; %—fractions; *p*—*p*-value.

**Table 2:** Asthma and allergy status characteristics (*n* = 37).

Characteristic	Results
The age of diagnosis of asthma	
*M* ± SD	6.4 ± 3.6
*Me* [*Q*1; *Q*3]	6 [4; 9]
*Min*–*Max*	1–13
Asthma duration	
*M* ± SD	4.6 ± 3.7
*Me* [*Q*1; *Q*3]	4 [1; 7]
*Min*–*Max*	0.5–12
Diagnosis of asthma before 6 years of age	17/37 (45.9%)
Other atopic diseases in the child	23/37 (62.2%)
Positive family history of atopic diseases	23/37 (62.2%)
Atopy	27/37 (73.0%)
Treatment with inhaled corticosteroids in the previous 4 weeks	30/37 (81.1%)
FEV 1%FVC (% predicted)	
*M* ± SD	89.3 ± 11.0
*Me* [*Q*1; *Q*3]	92 [83; 95]
*Min*–*Max*	64–116
FEV 1 (% predicted)	
*M* ± SD	96.5 ± 15.0
*Me* [*Q*1; *Q*3]	97 [91; 105]
*Min*–*Max*	57–130
FVC (% predicted)	
*M* ± SD	107.8 ± 15.0
*Me* [*Q*1; *Q*3]	108 [96; 113]
*Min*–*Max*	85–152

*M*—Mean; *SD*—Standard deviation; *Me*—Median (50%); *Q*1—Lower quartile (25%); *Q*3—Upper quartile (75%); *Min*—Minimum; *Max*—Maximum; *p*—*p*-value.

**Table 3:** Serum concentrations of L-ARG and its metabolites.

Serum L-ARG and Its Metabolite Concentration	Study Group *n* = 36	Control Group *n* = 26	*p*
L-ARG (μmol/L)			0.881
*M* ± SD	118.16 ± 28.32	121.81 ± 48.52	
*Me* [*Q*1; *Q*3]	115.0 [96.4; 132.7]	122.7 [90.1; 139.1]	
*Min*–*Max*	76.7–220.9	45.3–290.7	
ADMA (μmol/L)			0.304
*M* ± SD	0.56 ± 0.11	0.54 ± 0.18	
*Me* [*Q*1; *Q*3]	0.55 [0.50; 0.60]	0.52 [0.44; 0.60]	
*Min*–*Max*	0.35–0.81	0.28–1.18	
SDMA (μmol/L)			0.416
*M* ± SD	0.30 ± 0.07	0.32 ± 0.10	
*Me* [*Q*1; *Q*3]	0.29 [0.25; 0.36]	0.31 [0.27; 0.35]	
*Min*–*Max*	0.17–0.47	0.17–0.70	
CIT (μmol/L)			0.653
*M* ± SD	29.59 ± 8.69	28.45 ± 7.83	
*Me* [*Q*1; *Q*3]	28.1 [24.7; 33.5]	27.5 [24.3; 33.1]	
*Min*–*Max*	12.3–54.3	16.0–48.9	
ORN (μmol/L)			0.972
*M* ± SD	52.87 ± 20.38	63.40 ± 52.35	
*Me* [*Q*1; *Q*3]	45.5 [37.0; 70.0]	50.8 [38.0; 58.7]	
*Min*–*Max*	27.6–111.9	16.7–242.7	
DMA (μmol/L)			0.858
*M* ± SD	1.62 ± 0.67	1.67 ± 0.73	
*Me* [*Q*1; *Q*3]	1.6 [1.1; 2.1]	1.6 [1.0; 2.1]	
*Min*–*Max*	0.5–3.5	0.7–3.8	
ADMA/L-ARG ratio (%)			0.112
*M* ± SD	0.48 ± 0.12	0.49 ± 0.29	
*Me* [*Q*1; *Q*3]	0.5 [0.4; 0.5]	0.4 [0.4; 0.5]	
*Min*–*Max*	0.3–0.9	0.3–1.7	
SDMA/L-ARG ratio (%)			0.633
*M* ± SD	0.26 ± 0.07	0.29 ± 0.15	
*Me* [*Q*1; *Q*3]	0.3 [0.2; 0.3]	0.3 [0.2; 0.3]	
*Min*–*Max*	0.1–0.4	0.2–1.0	
DMA/L-ARG ratio (%)			0.960
*M* ± SD	1.40 ± 0.57	1.54 ± 1.11	
*Me* [*Q*1; *Q*3]	1.3 [1.0; 1.7]	1.3 [1.1; 1.6]	
*Min*–*Max*	0.5–2.7	0.6–6.5	

*M*—Mean; *SD* –Standard deviation; *Me*—Median (50%); *Q*1—Lower quartile (25%); *Q*3—Upper quartile (75%); *Min*—Minimum; *Max*—Maximum; *p*—*p*-value.

**Table 4:** Serum concentrations of IL-4.

Serum IL-4 Concentration	Study Group *n* = 37	Control Group *n* = 27	*p*
IL-4 (pg/mL)			0.716
*M* ± SD	0.16 ± 0.05	0.15 ± 0.04	
*Me* [*Q*1; *Q*3]	0.14 [0.13; 0.17]	0.14 [0.12; 0.16]	
*Min*–*Max*	0.10–0.34	0.11–0.28	

*M*—Mean; *SD*—Standard deviation; *Me*—Median (50%); *Q*1—Lower quartile (25%); *Q*3—Upper quartile (75%); *Min*—Minimum; *Max*—Maximum; *p*—*p*-value.

**Table 5:** EBC concentrations of L-ARG and its metabolites.

L-ARG and Its Metabolite Concentrations in EBC	Study Group *n* = 32	Control Group *n* = 20	*p*
L-ARG (μmol/L)			0.672
*M* ± SD	17.01 ± 29.38	16.48 ± 29.23	
*Me* [*Q*1; *Q*3]	5.9 [3,7; 10.6]	5.9 [4.5; 13.3]	
*Min*–*Max*	1.7–121.5	1.5–127.9	
ADMA (μmol/L)			0.323
*M* ± SD	0.31 ± 0.42	0.29 ± 0.40	
*Me* [*Q*1; *Q*3]	0.2 [0.2; 0.2]	0.2 [0.2; 0.2]	
*Min*–*Max*	0.1–1.9	0.1–1.9	
SDMA (μmol/L)			0.352
*M* ± SD	0.16 ± 0.08	0.14 ± 0.05	
*Me* [*Q*1; *Q*3]	0.1 [0.1; 0.1]	0.1 [0.1; 0.1]	
*Min*–*Max*	0.1–0.4	0.1–0.3	
CIT (μmol/L)			0.125
*M* ± SD	27.39 ± 34.99	37.77 ± 70.70	
*Me* [*Q*1; *Q*3]	15.9 [13.4; 20.3]	18.0 [16.0; 25.7]	
*Min*–*Max*	10.8–159.3	10.9–332.7	
ORN (μmol/L)			0.108
*M* ± SD	40.23 ± 65.02	80.73 ± 212.05	
*Me* [*Q*1; *Q*3]	15.7 [11.1; 29.5]	28.4 [15.3; 38.3]	
*Min*–*Max*	3.8–295.2	7.8–967.6	
DMA (μmol/L)			0.211
*M* ± SD	14.46 ± 6.48	17.13 ± 7.28	
*Me* [*Q*1; *Q*3]	14.2 [9.2; 16.8]	15.2 [11.6; 21.5]	
*Min*–*Max*	4.8–33.9	5.8–34.7	
ADMA/L-ARG ratio (%)			0.579
*M* ± SD	3.31 ± 1.80	3.17 ± 2.23	
*Me* [*Q*1; *Q*3]	3.0 [1.7; 4.3]	2.8 [1.6; 3.9]	
*Min*–*Max*	0.7–8.5	1.1–9.8	
SDMA/L-ARG ratio (%)			0.592
*M* ± SD	2.49 ± 1.68	2.32 ± 1.92	
*Me* [*Q*1; *Q*3]	2.2 [1.2; 3.5]	2.2 [1.0; 2.9]	
*Min*–*Max*	0.3–7.4	0.3–8.3	
DMA/L-ARG ratio (%)			0.843
*M* ± SD	274.29 ± 219.47	290.41 ± 231.87	
*Me* [*Q*1; *Q*3]	219.3 [112.3; 428.9]	203.2 [110.5; 420.4]	
*Min*–*Max*	8.1–764.7	15.6–795.0	

*M*—Mean; *SD* –Standard deviation; *Me*—Median (50%); *Q*1—Lower quartile (25%); *Q*3—Upper quartile (75%); *Min*—Minimum; *Max*—Maximum; *p*—*p*-value.

**Table 6:** Correlation of FEV1 and IL-4 in serum and EBC with L-ARG and its metabolites.

Parameter	L-ARG	ADMA	SDMA	CIT	ORN	DMA	ADMA/ L-ARG	SDMA/ L-ARG	DMA/ L-ARG
FEV1 and serum (*n* = 62) measurements	0.076	0.142	0.175	0.110	−0.022	0.109	−0.064	0.048	0.045
FEV1 and EBC (*n* = 52) measurements	−0.090	−0.151	−0.040	−0.202	−0.161	0.012	0.011	0.077	0.071
IL-4 and serum (*n* = 62) measurements	−0.014	0.039	0.109	0.213	0.160	0.080	0.065	0.082	0.136
IL-4 and EBC (*n* = 51) measurements	−0.050	−0.014	0.018	−0.053	0.080	0.102	0.064	0.038	0.088

Results are expressed as *Rho* values in Spearman’s rank correlation test.

## Data Availability

The data presented in this study are available on request from the corresponding author.

## Supplementary Materials

The following supporting information is available: https://www.mdpi.com/article/10.3390/jcm11010252/s1, Table S1: Serum concentrations of L-ARG, ADMA, SDMA, CIT, ORN, DMA in asthmatics aged 6–11 years and asthmatics aged 12–17 years. Table S2: EBC concentrations of L-ARG, ADMA, SDMA, CIT, ORN, DMA in asthmatics aged 6–11 years and asthmatics aged 12–17 years. Table S3: Spearman’s rank correlation coefficient for BMI and serum concentrations of L-ARG and its metabolites in asthmatics (*n* = 36). Table S4: Spearman’s rank correlation coefficient for BMI and EBC concentrations of L-ARG and its metabolites in asthmatics (*n* = 32). Table S5: Serum concentrations of L-ARG, ADMA, SDMA, CIT, ORN, DMA in atopic asthmatics and non-atopic asthmatics. Table S6: EBC concentrations of L-ARG, ADMA, SDMA, CIT, ORN, DMA in atopic asthmatics and non-atopic asthmatics. Table S7: Serum concentrations of L-ARG, ADMA, SDMA, CIT, ORN, DMA in asthmatics treated with inhaled corticosteroids in the previous 4 weeks and asthmatics who had no inhaled corticosteroids in the previous 4 weeks. Table S8: EBC concentrations of L-ARG, ADMA, SDMA, CIT, ORN, DMA in asthmatics treated with inhaled corticosteroids in the previous 4 weeks and asthmatics who had no inhaled corticosteroids in the previous 4 weeks.

## References

[1] Von Mutius E., Smits H.H. (2020). Primary prevention of asthma: From risk and protective factors to targeted strategies for prevention. Lancet.

[2] Faro A., Wood R.E., Schechter M.S., Leong A.B., Wittkugel E., Abode K., Chmiel J.F., Daines C., Davis S., Eber E. (2015). Official American Thoracic Society technical standards: Flexible airway endoscopy in children. Am. J. Respir. Crit. Care Med..

[3] Scott J.A., North M.L., Rafii M., Huang H., Pencharz P., Subbarao P., Belik J., Grasemann H. (2011). Asymmetric dimethylarginine is increased in asthma. Am. J. Respir. Crit. Care Med..

[4] Riccioni G., Bucciarelli V., Verini M., Consilvio N.P., Gallina S., Martini F., Aceto A., Scotti L., Bucciarelli T. (2012). ADMA, SDMA, L-Arginine and nitric oxide in allergic pediatric bronchial asthma. J. Biol. Regul. Homeost. Agents.

[5] Carraro S., Giordano G., Piacentini G., Kantar A., Moser S., Cesca L., Berardi M., Di Gangi I.M., Baraldi E. (2013). Asymmetric dimethylarginine in exhaled breath condensate and serum of children with asthma. Chest.

[6] Holguin F., Comhair S.A.A., Hazen S.L., Powers R.W., Khatri S.S., Bleecker E.R., Busse W.W., Calhoun W.J., Castro M., Fitzpatrick A.M. (2013). An association between L-arginine/asymmetric dimethyl arginine balance, obesity, and the age of asthma onset phenotype. Am. J. Respir. Crit. Care Med..

[7] Quinn K.D., Schedel M., Nkrumah-Elie Y., Joetham A., Armstrong M., Cruickshank-Quinn C., Reisdorph N., Gelfand E.W. (2017). Dysregulation of metabolic pathways in a mouse model of allergic asthma. Allergy.

[8] Prado C.M., Martins M.A., Tibério I.F. (2011). Nitric oxide in asthma physiopathology. ISRN Allergy.

[9] Bulau P., Zakrzewicz D., Kitowska K., Leiper J., Gunther A., Grimminger F., Eickelberg O. (2007). Analysis of methylarginine metabolism in the cardiovascular system identifies the lung as a major source of ADMA. Am. J. Physiol. Lung Cell Mol. Physiol..

[10] Barnes P.J. (1996). NO or no NO in asthma?. Thorax.

[11] Kharitonov S.A., Yates D., Robbins R.A., Logan-Sinclair R., Shinebourne E.A., Barnes P.J. (1994). Increased nitric oxide in exhaled air of asthmatic patients. Lancet.

[12] Lane C., Knight D., Burgess S., Franklin P., Horak F., Legg J., Moeller A., Stick S. (2004). Epithelial inducible nitric oxide synthase activity is the major determinant of nitric oxide concentration in exhaled breath. Thorax.

[13] Wells S.M., Buford M.C., Migliaccio C.T., Holian A. (2009). Elevated asymmetric dimethylarginine alters lung function and induces collagen deposition in mice. Am. J. Respir. Cell Mol. Biol..

[14] Wells S.M., Holian A. (2007). Asymmetric dimethylarginine induces oxidative and nitrosative stress in murine lung epithelial cells. Am. J. Respir. Cell Mol. Biol..

[15] Dweik R.A. (2007). The lung in the balance: Arginine, methylated arginines, and nitric oxide. Am. J. Physiol. Lung Cell Mol. Physiol..

[16] Van Den Berg M.P., Meurs H., Gosens R. (2018). Targeting arginase and nitric oxide metabolism in chronic airway diseases and their co-morbidities. Curr. Opin. Pharmacol..

[17] Maarsingh H., Zaagsma J., Meurs H. (2009). Arginase: A key enzyme in the pathophysiology of allergic asthma opening novel therapeutic perspectives. Br. J. Pharmacol..

[18] Horvath I., Hunt J., Barnes P.J., Alving K., Antczak A., Baraldi E., Becher G., van Beurden W.J., Corradi M., Dekhuijzen R. (2005). Exhaled breath condensate: Methodological recommendations and unresolved questions. Eur. Respir. J..

[19] Horváth I., Barnes P.J., Loukides S., Sterk P.J., Högman M., Olin A.-C., Amann A., Antus B., Baraldi E., Bikov A. (2017). A European Respiratory Society technical standard: Exhaled biomarkers in lung disease. Eur. Respir. J..

[20] Miller M.R., Hankinson J., Brusasco V., Burgos F., Casaburi R., Coates A., Crapo R., Enright P., Van Der Grinten C.P., Gustafsson P. (2005). Standardisation of spirometry. Eur. Respir. J..

[21] Bernstein I.L., Storms W.W. (1995). Practice parameters for allergy diagnostic testing. Joint Task Force on Practice Parameters for the Diagnosis and Treatment of Asthma. The American Academy of Allergy, Asthma and Immunology and the American College of Allergy, Asthma and Immunology. Ann. Allergy Asthma Immunol..

[22] Konstantinou G.N., Bousquet P.J., Zuberbier T., Papadopoulos N.G. (2010). The longest wheal diameter is the optimal measurement for the evaluation of skin prick tests. Int. Arch. Allergy Immunol..

[23] Fleszar M.G., Wiśniewski J., Zboch M., Diakowska D., Gamian A., Krzystek-Korpacka M. (2019). Targeted metabolomic analysis of nitric oxide/L-arginine pathway metabolites in dementia: Association with pathology, severity, and structural brain changes. Sci. Rep..

[24] Ahmad T., Mabalirajan U., Ghosh B., Agrawal A. (2010). Altered asymmetric dimethyl arginine metabolism in allergically inflamed mouse lungs. Am. J. Respir. Cell Mol. Biol..

[25] Lara A., Khatri S.B., Wang Z., Comhair S.A.A., Xu W., Dweik R.A., Bodine M., Levison B.S., Hammel J., Bleecker E. (2008). Alterations of the arginine metabolome in asthma. Am. J. Respir. Crit. Care Med..

[26] Kraj L., Krawiec M., Koter M., Graboń W., Kraj G., Chołojczyk M., Kulus M., Barańczyk-Kuźma A. (2014). Altered L-arginine metabolism in children with controlled asthma. Allergy Asthma Proc..

[27] Lau E.M., Morgan P.E., Belousova E.G., Toelle B.G., Ayer J.G., Celermajer D.S., Marks G.B. (2013). Asymmetric dimethylarginine and asthma: Results from the Childhood Asthma Prevention Study. Eur. Respir. J..

[28] MEDIVAC S.r.l. www.medivac.it/en/.

[29] Respiratory Research, Inc. https://respiratoryresearch.com/rtube/.

