# Supplementary material for: Metabolites of L-ARG in Exhaled Breath Condensate and Serum Are Not Biomarkers of Bronchial Asthma in Children
Source: J Clin Med. 2022 Jan 4;11(1):252. doi: 10.3390/jcm11010252 (PMC8746037; doi:10.3390/jcm11010252)
Supplement: Supplementary file 1 [file jcm-11-00252-s001.zip › jcm-1494741-supplementary.pdf]

Table S1: Serum concentrations of L-ARG, ADMA, SDMA, CIT, ORN, DMA in asthmatics aged 6-11 yr and asthmatics aged 12-17yr.

Serum L-ARG and its metabolites concentration	Age		p
	6–11 yr<br>n = 24	12–17 yr<br>n = 12	
L-ARG (µmol/L)			0.174
M ± SD	115.7 ± 32.0	123.0 ± 19.2	
Me [*Q*1; *Q*3]	111.5 [94.1; 127.8]	122,8 [105.8; 140.4]	
Min–Max	76.7–220.9	95.6–152.6	
ADMA (µmol/L)			0.275
M ± SD	0.58 ± 0.12	0.51 ± 0.09	
Me [*Q*1; *Q*3]	0.55 [0.50; 0.65]	0.55 [0.45; 0.57]	
Min–Max	0.39–0.81	0.35–0.60	
SDMA (µmol/L)			0,073
M ± SD	0,32 ± 0,07	0,27 ± 0,07	
Me [*Q*1; *Q*3]	0,30 [0,25; 0,36]	0,27 [0,21; 0,31]	
Min–Max	0.20–0.47	0.17–0.38	
CIT (µmol/L)			0.322
M ± SD	30.8 ± 9.6	27.1 ± 6.1	
Me [*Q*1; *Q*3]	28.1 [25.6; 34.6]	27.5 [21.3; 33.1]	
Min–Max	12.3–54.3	17.8–35.9	
ORN (µmol/L)			0.700
M ± SD	55.2 ± 22.8	48.2 ± 14.2	
Me [*Q*1; *Q*3]	48.9 [35.6; 73.9]	45.5 [41.1; 55.7]	
Min–Max	27.6–111.9	29.6–76.9	
DMA (µmol/L)			0.221
M ± SD	1.71 ± 0.71	1.42 ± 0.56	
Me [*Q*1; *Q*3]	1.72 [1.14; 2.13]	1.39 [0.99; 1.85]	
Min–Max	0.73–3.48	0.54–2.18	

M—Mean; *SD*—Standard deviation; *Me*—Median (50%); *Q*1—Lower quartile (25%); *Q*3—Upper quartile (75%); *Min*—Minimum; *Max*—Maximum; *p*—value.

Table S2: EBC concentrations of L-ARG, ADMA, SDMA, CIT, ORN, DMA in asthmatics aged 6-11 yr and asthmatics aged 12-17yr.

L-ARG and its metabolites concentrations in EBC	Age		*p*
	6 – 11yr *n* = 21	12 – 17 yr *n* = 11	
L-ARG (μmol/L)			0.812
M ± SD	20.4 ± 34.3	10.5 ± 16.0	
Me [Q1; Q3]	6.4 [3.5; 10.7]	5.9 [4.1; 6.8]	
Min–Max	1.7–121.5	2.2–58.1	
ADMA (μmol/L)			0.219
M ± SD	0.37 ± 0.51	0.20 ± 0.13	
Me [Q1; Q3]	0.17 [0.16; 0.22]	0.17 [0.15; 0.18]	
Min–Max	0.14–1.91	0.140.59	
SDMA (μmol/L)			0.968
M ± SD	0.17 ± 0.10	0.13 ± 0.01	
Me [Q1; Q3]	0.13 [0.13; 0.14]	0.13 [0.13; 0.13]	
Min–Max	0.12–0.45	0.12–0.18	
CIT (μmol/L)			0.905
M ± SD	29.0 ± 38.6	24.4 ± 28.3	
Me [Q1; Q3]	16.3 [13.4; 20.0]	15.5 [12.2; 21.9]	
Min–Max	10.8–159.3	11.3–109.0	
ORN (μmol/L)			0.190
M ± SD	44.7 ± 69.2	31.6 ± 58.4	
Me [Q1; Q3]	18.6 [13.3; 31.0]	13.8 [10.0; 25.1]	
Min–Max	4.9–295.2	3.8–206.4	
DMA (μmol/L)			0.104
M ± SD	15.9 ± 6.9	11.7 ± 4.5	
Me [Q1; Q3]	14.5 [11.8; 18.5]	13.9 [7.5; 15.7]	
Min–Max	6.3–33.9	4.8–16.7	

M—Mean; SD—Standard deviation; Me—Median (50%); Q1—Lower quartile (25%); Q3—Upper quartile (75%); Min—Minimum; Max—Maximum; *p*—value.

Table S3: Spearman's rank correlation coefficient for BMI and serum concentrations of L-ARG and its metabolites in asthmatics (*n* = 36).

	L-Arg	ADMA	SDMA	CYTR	ORN	DMA
rho	0.045	0.047	−0.052	−0.150	−0.018	0.053
p	0.789	0.781	0.759	0.375	0.917	0.754

There is no correlation between the analyzed parameters. All correlation coefficients do not differ from zero ($p > 0.05$).

Table S4: Spearman's rank correlation coefficient for BMI and EBC concentrations of L-ARG and its metabolites in asthmatics (*n* = 32).

	L-Arg	ADMA	SDMA	CYTR	ORN	DMA
rho	0.217	0.035	0.099	0.237	0.118	−0.009
p	0.228	0.845	0.583	0.187	0.399	0.961

There is no correlation between the analyzed parameters. All correlation coefficients do not differ from zero ($p > 0{,}05$).

Table S5: Serum concentrations of L-ARG, ADMA, SDMA, CIT, ORN, DMA in atopic asthmatics and non-atopic asthmatics.

Serum L-ARG and its metabolites concentration	Atopy status of asthmatics		p
	Atopic<br>n = 26	Non-atopic<br>n = 10	
L-ARG (µmol/L)			0.818
M ± SD	119.7 ± 31.0	114.2 ± 20.7	
Me [Q1; Q3]	113.4 [96.1; 138.7]	117.0 [99.0; 125.2]	
Min–Max	76.7–220.9	83.5–152.6	
ADMA (µmol/L)			0.659
M ± SD	0.55 ± 0.12	0.56 ± 0.10	
Me [Q1; Q3]	0.55 [0.50; 0.61]	0.56 [0.54; 0.59]	
Min–Max	0.35–0.81	0.39–0.74	
SDMA (µmol/L)			0.368
M ± SD	0.29 ± 0.08	0.31 ± 0.04	
Me [Q1; Q3]	0.28 [0.24; 0.36]	0.32 [0.27; 0.34]	
Min–Max	0.17–0.47	0.25–0.38	
CIT (µmol/L)			0.986
M ± SD	29.8 ± 9.6	28.9 ± 5.9	
Me [Q1; Q3]	28.1 [25.2; 33.5]	29.8 [24.3; 33.6]	
Min–Max	12.3–54.3	19.8–35.9	
ORN (µmol/L)			0.791
M ± SD	53.2 ± 18.8	51.9 ± 25.1	
Me [Q1; Q3]	47.0 [38.1; 71.9]	43.9 [35.9; 54.1]	
Min–Max	29.6–86.0	27.6–111.9	
DMA (µmol/L)			0.185
M ± SD	1.55 ± 0.73	1.78 ± 0.45	
Me [Q1; Q3]	1.42 [0.87; 2.02]	1.82 [1.59; 2.18]	
Min–Max	0.54–3.48	0.82–2.23	

M—Mean; *SD*—Standard deviation; *Me*—Median (50%); *Q1*—Lower quartile (25%); *Q3*—Upper quartile (75%); *Min*—Minimum; *Max*—Maximum; *p*—value.

Table S6: EBC concentrations of L-ARG, ADMA, SDMA, CIT, ORN, DMA in atopic asthmatics and non-atopic asthmatics.

L-ARG and its metabolites concentrations in EBC	Atopy status of asthmatics		*p*
	Atopic *n* = 25	Non-atopic *n* = 7	
L-ARG (µmol/L)			0.715
M ± SD	15.3 ± 25.0	22.9 ± 43.5	
Me [*Q*1; *Q*3]	5.9 [3.5; 10.5]	5.9 [4.3; 10.7]	
Min–Max	1.7–98.6	2.2–121.5	
ADMA (µmol/L)			0.523
68M ± SD	0.33 ± 0.46	0.26 ± 0.27	
Me [*Q*1; *Q*3]	0.17 [0.16; 0.21]	0.17 [0.15; 0.17]	
Min–Max	0.14–1.91	0.14–0.88	
SDMA (µmol/L)			0.553
M ± SD	0.15 ± 0.07	0.17 ± 0.12	
Me [*Q*1; *Q*3]	0.13 [0.13; 0.14]	0.13 [0.13; 0.13]	
Min–Max	0.12–0.38	0.12–0.45	
CIT (µmol/L)			0.820
M ± SD	29.7 ± 39.1	19.0 ± 9.9	
Me [*Q*1; *Q*3]	15.5 [13.3; 20.6]	16.2 [13.8; 18.9]	
Min–Max	10.8–159.3	11.3–40.7	
ORN (µmol/L)			0.438
M ± SD	43.7 ± 71.1	27.9 ± 37.6	
Me [*Q*1; *Q*3]	18.2 [12.3; 28.1]	14.7 [10.0; 31.0]	
Min–Max	4.9–295.2	3.8–111.0	
DMA (µmol/L)			0.171
M ± SD	13.7 ± 6.1	17.2 ± 7.6	
Me [*Q*1; *Q*3]	12.9 [9.0; 16.5]	16.3 [13.7; 23.1]	
Min–Max	6.3–33.9	4.8–29.3	

M—Mean; *SD*—Standard deviation; *Me*—Median (50%); *Q*1—Lower quartile (25%); *Q*3—Upper quartile (75%); *Min*—Minimum; *Max*—Maximum; *p*—value.

Table S7: Serum concentrations of L-ARG, ADMA, SDMA, CIT, ORN, DMA in asthmatics treated with inhaled corticosteroids in the previous 4 weeks and asthmatics who had no inhaled corticosteroids in the previous 4 weeks

Serum L-ARG and its metabolites concentration	Treatment with inhaled corticosteroids in the previous 4 weeks		*p*
	Yes<br>*n* = 29	No<br>*n* = 7	
ADMA (µmol/L)			0.905
M ± SD	0.55 ± 0.11	0.56 ± 0.12	
Me [*Q*1; *Q*3]	0.6 [0.5; 0.6]	0.5 [0.5; 0.6]	
Min–Max	0.4–0,8	0.4–0,8	
SDMA (µmol/L)			0,201
M ± SD	0.29 ± 0.07	0.34 ± 0.08	
Me [*Q*1; *Q*3]	0.3 [0.2; 0.3]	0.3 [0.3; 0.4]	
Min–Max	0.2–0.4	0.2–0.5	
CIT (µmol/L)			0.576
M ± SD	29.27 ± 9.21	30.91 ± 6.47	
Me [*Q*1; *Q*3]	27.4 [22.6; 33.5]	29.9 [25.4; 36.1]	
Min–Max	12.3–54.3	24.3–41.9	
ORN (µmol/L)			0.093
M ± SD	50.3 ± 20.2	63.6 ± 18.9	
Me [*Q*1; *Q*3]	42.4 [35.9; 61.0]	61.7 [50.0; 82.5]	
Min–Max	27.6–111.9	31.7–86.0	
DMA (µmol/L)			0.379
M ± SD	1.56 ± 0.64	1.85 ± 0.77	
Me [*Q*1; *Q*3]	1.6 [1.1; 2.0]	2.0 [1.2; 2.5]	
Min–Max	0.5–3.5	0.8–3.0	

M—Mean; SD—Standard deviation; Me—Median (50%); Q1—Lower quartile (25%); Q3—Upper quartile (75%); Min—Minimum; Max—Maximum; *p*—value.

Table S8: EBC concentrations of L-ARG, ADMA, SDMA, CIT, ORN, DMA in asthmatics treated with inhaled corticosteroids in the previous 4 weeks and asthmatics who had no inhaled corticosteroids in the previous 4 weeks.

L-ARG and its metabolites concentrations in EBC	Treatment with inhaled corticosteroids in the previous 4 weeks		*p*
	Yes<br>*n* = 26	No<br>*n* = 6	
L-ARG (μmol/L)			0.398
M ± SD	17.1 ± 29.8	16.6 ± 30.3	
Me [*Q*1; *Q*3]	6.3 [4.1; 10.7]	4.7 [3.2; 5.9]	
Min–Max	1.7–121.5	2.6–78.4	
ADMA (μmol/L)			0.257
M ± SD	0.28 ± 0.34	0.45 ± 0.71	
Me [*Q*1; *Q*3]	0.17 [0.16; 0.21]	0.15 [0.15; 0.18]	
Min–Max	0.14–1.76	0.14–1.91	
SDMA (μmol/L)			0.981
M ± SD	0.15 ± 0.08	0.17 ± 0.10	
Me [*Q*1; *Q*3]	0.13 [0.13; 0.14]	0,13 [0.13; 0.13]	
Min–Max	0.12–0.45	0.12–0.38	
CIT (μmol/L)			0.904
M ± SD	24.6 ± 28.0	39.5 ± 58.8	
Me [*Q*1; *Q*3]	15.3 [13.4; 20.0]	16.3 [12.5; 22.0]	
Min–Max	11.3–125.4	10.8–159.3	
ORN (μmol/L)			0.646
M ± SD	40.4 ± 66.8	39.5 ± 62.7	
Me [*Q*1; *Q*3]	15.7 [12.0; 30.9]	16.1 [9.2; 25.1]	
Min–Max	4.9–295.2	3.8–166.6	
DMA (μmol/L)			0.546
M ± SD	14.8 ± 6.3	13.0 ± 7.5	
Me [*Q*1; *Q*3]	14.2 [9.8; 16.7]	12.0 [6.3; 20.1]	
Min–Max	6.5–33.9	4.8–22.7	

M—Mean; SD—Standard deviation; Me—Median (50%); Q1—Lower quartile (25%); Q3—Upper quartile (75%); Min—Minimum; Max—Maximum; *p*—value.
